# Treatment strategies for low-risk papillary thyroid carcinoma: a position statement from the Thyroid Department of the Brazilian Society of Endocrinology and Metabolism (SBEM)

**DOI:** 10.20945/2359-3997000000512

**Published:** 2022-09-08

**Authors:** Laura Sterian Ward, Rafael Selbach Scheffel, Ana O. Hoff, Carolina Ferraz, Fernanda Vaisman

**Affiliations:** 1 Universidade Estadual de Campinas Faculdade de Ciências Médicas Laboratório de Genética Molecular do Câncer Campinas SP Brasil Laboratório de Genética Molecular do Câncer, Faculdade de Ciências Médicas, Universidade Estadual de Campinas (Unicamp), Campinas, SP, Brasil; 2 Universidade Federal do Rio Grande do Sul Faculdade de Medicina Hospital de Clínicas de Porto Alegre Porto Alegre RS Brasil Unidade de Tireoide, Hospital de Clínicas de Porto Alegre, Faculdade de Medicina, Universidade Federal do Rio Grande do Sul, Porto Alegre, RS, Brasil; 3 Universidade Federal do Rio Grande do Sul Instituto de Ciências Básicas da Saúde Departamento de Farmacologia Porto Alegre RS Brasil Departamento de Farmacologia, Instituto de Ciências Básicas da Saúde, Universidade Federal do Rio Grande do Sul, Porto Alegre, RS, Brasil; 4 Universidade de São Paulo Instituto do Câncer do Estado de São Paulo (Icesp) Unidade de Oncologia Endócrina São Paulo SP Brasil Unidade de Oncologia Endócrina, Instituto do Câncer do Estado de São Paulo (Icesp), Universidade de São Paulo (USP), São Paulo, SP, Brasil; 5 Irmandade da Santa Casa de Misericórdia de São Paulo Faculdade de Ciências Médicas da Santa Casa Departamento de Medicina São Paulo SP Brasil Divisão de Endocrinologia, Departamento de Medicina, Irmandade da Santa Casa de Misericórdia de São Paulo, São Paulo, SP, Brasil; Faculdade de Ciências Médicas da Santa Casa, São Paulo, SP, Brasil; Faculdade de Ciências Médicas da Santa Casa São Paulo SP Brasil; 6 Instituto Nacional do Câncer do Rio de Janeiro Serviço de Oncologia Endócrina Rio de Janeiro RJ Brasil Serviço de Oncologia Endócrina, Instituto Nacional do Câncer do Rio de Janeiro (Inca), Rio de Janeiro, RJ, Brasil; 7 Universidade Federal do Rio de Janeiro Faculdade de Medicina Rio de Janeiro RJ Brasil Faculdade de Medicina, Serviço de Endocrinologia, Universidade Federal do Rio de Janeiro, Rio de Janeiro, RJ, Brasil

**Keywords:** Papillary thyroid carcinoma, position statement, active surveillance

## Abstract

Increasingly sensitive diagnostic methods, better understanding of molecular pathophysiology, and well-conducted prospective studies have changed the current approach to patients with thyroid cancer, requiring the implementation of individualized management. Most patients with papillary thyroid carcinoma (PTC) are currently considered to have a low risk of mortality and disease persistence/recurrence. Consequently, current treatment recommendations for these patients include less invasive or intensive therapies. We used the most recent evidence to prepare a position statement providing guidance for decisions regarding the management of patients with low-risk PTC (LRPTC). This document summarizes the criteria defining LRPTC (including considerations regarding changes in the TNM staging system), indications and contraindications for active surveillance, and recommendations for follow-up and surgery. Active surveillance may be an appropriate initial choice in selected patients, and the criteria to recommend this approach are detailed. A section is dedicated to the current evidence regarding lobectomy versus total thyroidectomy and the potential pitfalls of each approach, considering the challenges during long-term follow-up. Indications for radioiodine (RAI) therapy are also addressed, along with the benefits and risks associated with this treatment, patient preparation, and dosage. Finally, this statement presents the best follow-up strategies for LRPTC after lobectomy and total thyroidectomy with or without RAI.

## INTRODUCTION

We have witnessed over the last decade a paradigm shift in terms of staging and management of patients with papillary thyroid carcinoma (PTC) from a standardized approach to individualized assessment and treatment. These changes have occurred mostly due to the development of risk assessment tools that have allowed early prediction during follow-up of meaningful outcomes such as disease-specific mortality and risks of structural disease persistence/recurrence or therapeutic failure. The approach to individualized PTC management is built on the importance of tumor histology, quality of the first surgery, serum thyroglobulin (Tg) levels in the postoperative evaluation, and decisions regarding the choices for the initial therapy and adjuvant therapy with radioactive iodine (RAI) (1). Based on this approach, most patients with PTC are classified as having a low risk of mortality and persistence/recurrence disease and, for this reason, the current recommendations include less invasive or intensive therapies (2,3).

### Assessing the mortality risk

Death is usually the primary concern of patients with cancer during the first office appointment. Hence, an accurate classification to predict cancer-specific mortality is crucial. As with other solid tumors, the American Joint Committee on Cancer (AJCC) staging system, known as TNM (tumor, node, and metastasis), is widely used in PTC. This staging system considers the tumor size and local invasion by the primary tumor and the presence of metastatic lymph nodes and distant metastases, all divided by age group. The AJCC/TNM system performs well compared with other staging classifications and is the most used system in tumor registries worldwide. The recent AJCC 8^th^ edition has brought a few changes that seem to enhance the accuracy of the prediction of disease-specific mortality (4,5). Three main changes are worth mentioning. First, the cutoff age at diagnosis increased from 45 to 55 years. This change was supported by an international multicenter study with almost 10,000 patients showing that the age of 55 years was better at separating patients with stages III versus IV disease (6). Second, the AJCC 8^th^ edition reevaluated the prognostic significance of minor extrathyroidal extension (mETE), defined as subclinical perithyroidal invasion only detectable on histology (*i.e.*, not apparent on intraoperative inspection or imaging evaluation). The prior AJCC 7^th^ edition had classified any tumor with mETE as T3, but studies have shown that mETE alone has no influence on disease-free survival or disease-specific survival, despite conflicting data regarding its influence on persistence/recurrence of disease in thyroid bed or cervical lymph nodes. Additionally, a recent study by Tam and cols. has suggested that tumor size is an independent predictor of those outcomes (7). Considering these factors, the AJCC 8^th^ edition has subdivided the T3 classification into T3a for tumors above 4 cm confined to the thyroid gland and T3b for tumors with ETE defined as gross strap muscle invasion. The third change in the AJCC 8^th^ edition that is worth mentioning is regarding mediastinal metastatic lymph nodes, referred to as level VII lymph nodes. Previously, N1a was used only when metastatic lymph nodes were found in level VI (central compartment, *i.e.*, pretracheal, paratracheal, or prelaryngeal). Now, metastases to these mediastinal lymph nodes are categorized as N1a, while N1b is used for metastatic lymph nodes in the lateral neck (8,9). Additionally, older patients with N1 disease are no longer upstaged to stage III or IV disease (2); patients with N1 disease who are younger or older than 55 years are now classified as having stage I or II disease, respectively.

Multiple publications have demonstrated that, compared with the AJCC 7^th^ edition, the 8th edition downstages a substantial number of low-risk patients and provides better separation between stage groups (7,9,10). Most (>90%) patients are categorized into stages I and II and have a risk of cancer-related death below 1% (11).

### Assessing disease persistence/recurrence

Considering that patients with DTC have very low mortality rates, assessing these patients’ risk based on disease persistence/recurrence seems to be more adequate. Hence, stratification based on histopathological features, presence of lymph node and distant metastases, and some postoperative information such as response to therapy has been endorsed (5). Based on these considerations, the American Thyroid Association (ATA) risk-stratification system has been widely used. This system classifies patients into the following three categories of disease recurrence or persistence (2): low-risk (<5%), intermediate-risk (5%-20%), and high-risk (>20%). This risk assessment is currently based on histopathological features of the tumor and will likely include molecular features in the near future. Additionally, operative findings (not described in the pathological report) including vocal cord paralysis, extent of gross extrathyroidal invasion, and completeness of resection, should be considered in the risk assessment. Patients with low-risk disease encompass 57% of all patients with PTC (12). This category of low-risk disease includes (2):

- Patients with very low risk of recurrence (< 1%, unifocal, tumor size < 1 cm if PTC),
- Patients with PTC with intrathyroidal tumors < 4 cm, without clinical evidence of lymph node metastases or with ≤ 5 microscopic (< 2 mm) lymph node metastases on histology, without vascular invasion, and without an aggressive variant of PTC, and
- Patients with well-differentiated follicular thyroid cancer with capsular invasion alone or with less than four foci of vascular invasion.

Molecular characterization of the tumor has not been adopted routinely yet, and the impact of *BRAF* or other (*e.g., TERT*) mutations on risk assessment is still a matter of debate (13,14).

### Definition of low-risk papillary thyroid carcinoma

A better understanding of the prognostic tools in PTC has clarified the favorable course of patients with low-risk disease and opened the discussion about the best approach in such cases. The management options for these patients have expanded, ranging from simple observation (active surveillance [AS]) to total thyroidectomy (TT) with or without adjuvant RAI therapy. These decisions must be individualized and adapted according to the resources available at each center.

Given these considerations, we present herein the recommendations of the Brazilian Society of Endocrinology and Metabolism regarding the management of low-risk PTC (LRPTC) based on the ATA 2015 guidelines (2). **Depending on age, tumor size with no extrathyroidal invasion, and lymph node status (less than 5 AND all less than 0,2 cm), patients classified as having AJCC/TNM stage I or II will be considered to be at low risk for structural disease recurrence and disease-specific death**.

## ACTIVE SURVEILLANCE

The strategy of AS was initially recommended for the management of microPTC. This strategy prevents excessive early treatment, among other advantages. Monitoring of microPTC gained recognition in 2010 when a pioneer group published the first data showing safe follow-up of patients with microPTC managed without intervention and in whom the tumor was clearly visualized on ultrasonography (US) (15).

**Figure 1:**
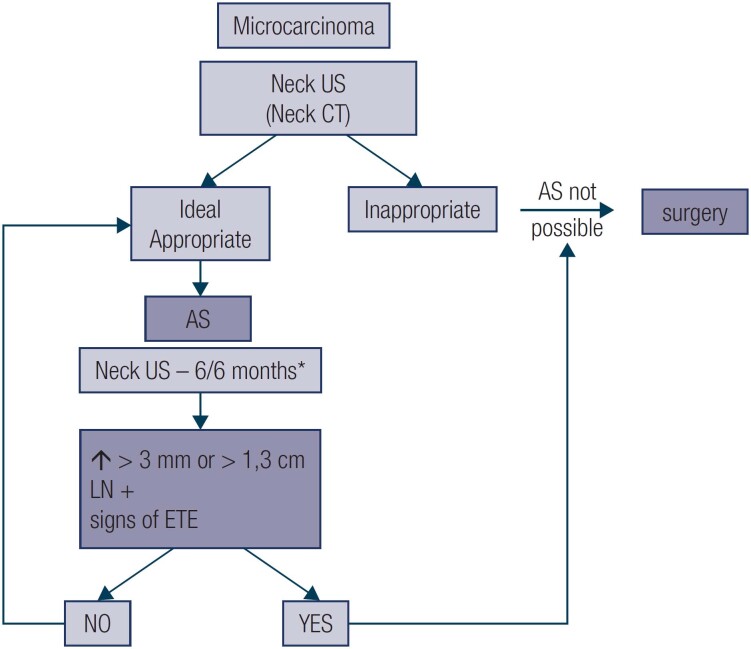
Follow-up and recommendation for surgery during active surveillance. wid *Every 6 months in the first 2 years and annually thereafter. Abbreviations: AS, active surveillance; US, ultrasonography; CT, computed tomography; LN, lymph node; ETE, extrathyroidal extension.

Guidelines recommend AS as the initial approach in selected patients with microPTC (2,16–18). In addition to recommending AS for lesions < 1 cm, the ATA guidelines also recommend this approach to low-risk, completely intrathyroidal tumors measuring up to 1.5 or 2 cm (2). Several studies from different countries have confirmed initial data from Japan that has shown nodular growth in approximately 10% of the patients with microPTC and lymph node metastasis in less than 5% of them, with none of the studies describing distant metastases or death during AS (19–24). **Fewer studies have included tumors measuring up to 1.5 or 2.0 cm tumors (****2****);** **Figure 1** **includes microPTC as we need more data regarding larger tumors.**

### Indications and contraindications for active surveillance

Before a patient is started on AS, three main points must be evaluated, *i.e.*, the characteristics of the nodule, characteristics of the patient, and characteristics of the team that will follow the patient. This initial analysis will identify if the patient is an ideal, appropriate, or inappropriate candidate for AS (25) (Table 1). Patients considered to be an ideal candidate for AS in this initial analysis and who fulfilled all criteria should be offered AS along with the information that future surgery may be necessary if the nodule grows in size or signs of (lymph nodes or distant) metastases emerge, or at the patient’s discretion. In contrast, patients who do not fit into any of the three categories should be referred for thyroidectomy.

**Table 1:** Patient classification as ideal, appropriate, and inappropriate to initiate AS

	Ideal	Appropriate	Inappropriate
Nodule characteristics	Solitary nodule Well-defined margisn cN0, cM0 No extrathyroidal extension	Multifocal Subcapsular location (not adjacent to RLN) Background US findings	Aggressive cytology Subcapsular location adjacent to RLN Obtuse angles between tumors and the trachea cN1, cM1 Evidence of extrathyroidal extension
Patient characteristics	>60 years Acceptance (also family members) Follow-up plans Life-threatening comorbidities “Minimalistic”	18 to 59 years Strong family history of PTC Childbearing potential	<18 years Unlikely to make follow-up No acceptance “Maximalist”
Medical team characteristics	Experienced multidisciplinary team High quality US Prospective data collection Reminder program to ensure follow-up	Experienced endocrinologist/surgeon US routinely available	US not available Little experience with thyroid cancer management

RLN: recurrent laryngeal nerve; US: ultrasound; PTC: papillary thyroid carcinoma.

Modified from: Brito and cols. (25).

Of note, these nodules have an approximate 50% chance of growth over 10 years in patients younger than 40 years, particularly in those closer to the age of 20 years (26,27). Still, the prognosis is not affected if AS is chosen for these patients and future surgery is required due to nodular growth (28).

### Special situations

#### Family history

Familial nonmedullary thyroid carcinoma has a prevalence of around 3%-10% and is defined as the presence of nonmedullary thyroid carcinoma in at least three first-degree relatives in the absence of other known familial syndromes (29). Although some studies have shown that the risk of recurrence or mortality is comparable in cases of familial versus sporadic nonmedullary thyroid carcinoma, familial cases appear to have an increased risk of multifocal lesions (30–32). Although AS may be a therapeutic option in confirmed cases of familial microPTC, surgery is a better choice and TT should be recommended in these cases (28).

### Childbearing age or pregnancy

Despite a few reports associating levels of beta human chorionic gonadotropin (hCG) with increased number and volume of thyroid nodules during pregnancy (33,34), the aggressiveness of these tumors is not increased (35). Therefore, women of childbearing age or pregnant who are diagnosed with microPTC may also undergo AS following the same criteria shown in Table 1, **however, more studies with long follow up are needed in pregnant women and those who want to become pregnant.**

### Concomitant thyroid disorders (Graves’ disease, Hashimoto’s thyroiditis, associated benign nodules)

Although evidence shows an increased risk of thyroid cancer in patients with autoimmune diseases (36), AS is currently not contraindicated in patients with Graves’ disease or Hashimoto’s thyroiditis (28,36). However, two aspects must be kept in mind regarding these patients. The first is that they should be maintained euthyroid to prevent stimulation of nodular growth due to high TSH values, and the second is to assess whether the heterogeneity of the thyroid on US could impair the measurement and proper follow-up of the tumor (2).

Regarding the presence of associated benign nodules, treatment is not required when these nodules are not clinically significant (*e.g.*, toxic nodules or bulky nodules leading to compressive symptoms) (2,28).

### TSH suppression therapy during active surveillance

Evidence of benefits from levothyroxine suppressive therapy in patients undergoing AS remains inconclusive since no randomized trials have been conducted to evaluate this effect. At the moment, the safest strategy to prevent nodular growth and deleterious effects from excessive thyroid hormone (especially in elderly patients) is to maintain TSH values within the normal range (2,28).

### Follow-up and indications for surgery

Patients undergoing AS must be reassessed at each visit, with a focus on the characteristics of the nodule and the ability of the patient and the staff to continue AS (Figure 1). Neck CT may be helpful in cases of nodules close to or in contact with the trachea in order to better visualize the contact angle between them. An obtuse angles between tumors and the trachea should be indication for surgery (Table 1).

Indications for surgery during AS include a > 3 mm growth in tumor size, growth of the tumor to a diameter ≥ 13 mm, detection of lymph node metastasis, diagnosis of other thyroid or parathyroid disease, or a change in the patient’s therapeutic preference. **Based on current recommendations, clinical and US assessments and appointments should be performed every 6 months for the first 2 years and annually thereafter, if no clinical or ultrassonographic changes, have been suggested** (25,28).

### Special considerations before the implementation of active surveillance in Brazil

Before AS is implemented at a center, some points must be considered to ensure that the patients will be safely followed up:

1. The medical team that will follow the patient must be experienced in managing thyroid cancer. Additionally, within the team and between the team and the other doctors caring for the patient there must be a consensus regarding the patient’s management.
2. The medical team must be prepared at all stages of follow-up to clarify the patients’ concerns regarding treatment indications and contraindications, reassure the patients and help them overcome their fears, and be available to talk to the patients and their families.
3. The infrastructure of the center should facilitate the patient’s follow-up, ensuring the availability of appointments within the specified follow-up period. The center must also ensure the availability of quality US during follow-up or, when possible, offer quality US within its premises.
4. Centers without experience with AS should choose to follow the patient under a research protocol.

## SURGICAL TREATMENT

### Lobectomy versus total thyroidectomy

The choice between TT (with or without RAI therapy) versus lobectomy has little impact on recurrence rates or risk of death from thyroid cancer. Solid evidence has shown that simple observation of the contralateral lobe is safe in patients with low-risk disease after lobectomy (2,37,38).

### Indications for lobectomy versus total thyroidectomy

Partial thyroidectomy (PT) or lobectomy are considered to be sufficient in treating T1 and T2 tumors confined to the thyroid according to the ATA consensus, considering that the extent of the thyroidectomy has no effect on survival in PTC (39) and that recurrence after lobectomy is salvageable without a negative impact on the overall survival (2). Therefore, in our opinion, either PT or lobectomy is a good option for patients in Brazil, especially considering the limited number of head and neck surgeons with high surgical volume in the country. Notably, a systematic review and meta-analysis including 13,801 patients with microPTC (8,812 submitted to TT and 4,989 to PT) from 11 different cohorts has concluded that partial TT had equivalent results in terms of mortality, which is relatively low in these cases (40).

The maintenance of thyroid tissue in PT avoids the need for thyroid hormone replacement that inevitably results from a TT. In addition to an increased somatic and psychiatric burden, hypothyroidism may lead to a substantial socioeconomic impact in the form of early retirement or loss of income (41). However, even after lobectomy the need for hormone replacement is not uncommon, and half of the patients undergoing hemithyroidectomy may develop hypothyroidism (42). Beyond that, Tg levels may be more conveniently followed up after TT (43,44).

Completion thyroidectomy may be required in cases of persistent or recurrent disease. After lobectomy, a histopathological report showing evidence of lymph node or distant metastasis, or an increased number of risk factors for recurrence (such as ETE, aggressive variants, reported positive margins either by pathologist or gross disease invasion reported by the surgeon, extensive angioinvasion, or lymphovascular or neural invasion) should prompt completion thyroidectomy and RAI treatment for a more favorable follow-up. The optimal time for completion thyroidectomy with fewer complications has been suggested to be 3 months (45,46).

In summary, TT allows for Tg levels to be more conveniently followed up (especially when RAI is used) and may decrease recurrence rates but increases morbidity. In contrast, lobectomy is a less extensive surgery that preserves some intrinsic thyroid function. Recent evidence suggests that lobectomy is a cost-effective strategy in middle-aged patients with LRPTC. In contrast, AS is cost-effective beginning at the age of 69 years (47). Hence, the decision regarding the extent of the surgical treatment in LRPTC must involve both the surgeon and the endocrinologist and consider the patients’ beliefs, socioeconomic and cultural characteristics, and access to adequate health services. Table 2 summarizes the current evidence that may guide choices (48).

**Table 2:** Summary of the main advantages and disadvantages of partial thyroidectomy in comparison to total thyroidectomy

Advantages of lobectomy versus total thyroidectomy	Disadvantages of lobectomy versus total thyroidectomy
Lower surgical risks	Risk of completion surgery to improve prognosis and/or administer radioactive iodine
Thyroid hormone supplementation may not be necessary	Patients may still require thyroid hormone supplementation (thyroiditis or little remaining parenchyma)
Comparable survival	No evidence of quality of life improvement
Completion surgery, if necessary, does not increase surgical risk neither modify outcome	Not adequate for intermediate- and high-risk patients
	Thyroglobulin may not be appropriate for follow-up

Modified from: Hartl DM, Guerlain J, Breuskin I, Hadoux J, Baudin E, Al Ghuzlan A, Terroir-Cassou-Mounat M, Lamartina L, Leboulleux S. Thyroid Lobectomy for Low to Intermediate Risk Differentiated Thyroid Cancer. Cancers (Basel). 2020 Nov 6;12(11):3282.

## RADIOACTIVE IODINE TREATMENT

Postoperative RAI must be considered only in patients treated with TT and with the following goals (2,3):

1. Ablation (destruction of remaining thyroid tissue, which can improve the sensitivity of serum Tg measured during follow-up),
2. Adjuvant treatment (destruction of microscopic occult disease to reduce risk of recurrence),
3. Treatment (destruction of persistent or metastatic disease to improve disease-free survival or overall survival), and
4. Improved staging with post-treatment **Whole body scan** (WBS).

### Radioactive iodine for remnant ablation

Previously, the role of remnant ablation was to facilitate postoperative follow-up and initial staging with measurement of stimulated Tg level and post-treatment WBS (12). However, with the advent of highly sensitive Tg assays and US, patients with LRPTC can be safely followed up without ablation of the normal thyroid tissue. After surgery, both serum Tg and cervical US can predict long-term prognosis and help decide whether to proceed or not with ablation (48). Patients with low-risk DTC and without Tg antibodies (TgAb) who present with normal neck US and serum Tg levels < 0.2 ng/mL on TSH suppression or < 1 ng/mL after recombinant TSH stimulation require no ablation (49,50). Notably, the performance and clinical impact of WBS have been questioned, and recent data have shown that this procedure has little impact on staging, especially in patients with low-risk or intermediate-risk disease (51,52). Furthermore, Janovsky and cols. also showed that in low risk patients, overtime, Tg levels usually decline without radioiodine ablation, suggesting that Tg trend can also be used to avoid unnecessary treatment in these patients, especially with negative post-operative ultrasound (53). Recently, a large prospective study performed in France (ESTIMABL 2) showed that in patients with low-risk thyroid cancer undergoing thyroidectomy, a follow-up strategy that did not involve the use of radioiodine was noninferior to an ablation strategy with radioiodine regarding the occurrence of functional, structural, and biologic events at 3 years (54).

Ablation can be considered in patients with detectable TgAb levels and in those with increased postoperative Tg levels in the context of a normal cervical US. Notably, multivariate analyses from several studies have shown postoperative serum Tg level to be an independent predictor of disease recurrence (55–57).

### Radioactive iodine as adjuvant treatment

The decision to proceed with adjuvant RAI therapy should be based on the risks and benefits of RAI in patients with low risk of recurrence and on the fact that delayed detection of the disease does not reduce survival. Currently, the decision and recommendation for RAI treatment in this group of patients are based mostly on retrospective studies indicating no substantial effect of adjuvant RAI therapy on overall or disease-free survival (12,55) and on prospective data from the NTCTCSG registry in which multivariate analyses demonstrated no significant effect of RAI treatment in low-risk patients (56,57). The results from two undergoing prospective trials (ESTIMABL2 [NCT01837745] and IoN [NCT01398085] trials) in which patients are randomized to RAI versus no RAI therapy will be able to confirm the impact on overall or disease-free survival of postoperative adjuvant RAI in low-risk patients. In summary, based on current evidence, adjuvant RAI treatment is not routinely recommended for risk reduction or with adjuvant intent in patients with a low risk of recurrence (2).

### Preparation and recommended radioiodine activity

Considering that the recurrence rates are low and a delayed diagnosis of persistent or recurrent disease has no effect on survival, it is important to provide the lowest RAI activity to avoid unnecessary risks from radiation exposure. Several studies indicate that treatments with a RAI dose of 1.1 GBq (30 mCi) provide the same results as those with a dose of 3.7 GBq (100 mCi) (47,58-62). Additionally, studies comparing the preparation for RAI therapy using thyroid hormone withdrawal versus recombinant TSH administration have shown both strategies to be effective, although the use of recombinant TSH has been associated with better quality of life (49,60,61). Therefore, when RAI therapy is recommended, the preparation can be performed with recombinant TSH (when available), and the RAI dose can be low (1.1 GBq) and should be administered after 2 weeks of a low-iodine diet.

## FOLLOW-UP STRATEGIES

The main goal of long-term follow-up in PTC is to detect disease recurrence in patients classified as being disease-free and progression in those with persistent disease. A second objective is to adequately manage the consequences of the initial treatment, including hypothyroidism and hypoparathyroidism. The risk of disease recurrence/persistence is low in LRPTC, as stated above, and the follow-up of patients with LRPTC differ according to the surgical treatment and the use or not of RAI (63). Patients on AS have a specific follow-up protocol, as described above.

The initial risk of recurrent/persistent disease should be refined during follow-up in light of the patient’s response to therapy 6-18 months after the initial treatment. It should be predominantly based on Tg level, detection of TgAb, and imaging findings (dynamic risk stratification). If the imaging study reveals persistent tumor foci, the patient’s response to treatment is classified as structurally incomplete. Conversely, if the imaging examination is negative, the response to treatment is classified as excellent if Tg and TgAb levels are undetectable, indeterminate if serum Tg levels are low, or biochemically incomplete if Tg levels are high (Table 3) (64).

**Table 3:** Risk Stratification based on response to therapy 6-24 months after initial treatment (total thyroidectomy + RAI)

Response to Therapy	Definitions	
Excellent Response	No clinical, biochemical or structural evidence of disease	Nonstimulated Tg <0.2 ng/mL and/or Stimulated Tg <1 ng/mL + Undetectable TgAb + Negative imaging
Indeterminate Response	Non-specific biochemical or structural findings which cannot be confidently classified as either benign or malignant	Nonstimulated 0.2-1 ng/mL and/or Stimulated Tg 1-10 ng/mL and/or TgAb levels stable or declining and/or Nonspecific findings on imaging studies
Biochemical Incomplete Response	Abnormally elevated serum Tg and/or increasing Tg values over time with similar TSH levels and/or rising TgAb levels in the absence of localizable structural disease	Nonstimulated >1 ng/mL and/or Stimulated Tg >10 ng/mL and/or Increasing TgAb + Negative imaging
Structural Incomplete Response	Persistent or newly identified loco-regional or distant metastases with or without abnormal Tg or TgAb	Structural evidence of disease in anatomic (US, CT), functional (WBS) or hybrid (^18^FDG-PT/CT) imaging regardless of Tg or TgAb values

Adapted from: Haugen and cols. (2) Tuttle and cols. (11) Momesso DP and cols. (67).

Tg: thyroglobulin; TgAb: thyroglobulin antibodies.

### Follow-up after lobectomy

The follow-up of patients treated with lobectomy is mainly based on the results of neck US. The emergence of benign nodules during follow-up (which occurs in 20%-50% of the patients) is far more common than tumor recurrence, which occurs in about 5% of all patients after lobectomy (37,65,66).

The proposed cutoff values for Tg in the assessment of treatment response are reported in Table 3. The temporal trend of Tg levels is more important than isolated Tg levels and can be a valuable guide during follow-up (44). A recent study of 1451 patients with PTC who underwent lobectomy has shown that postoperative Tg levels are reasonably valuable for surveillance. A Tg value of 5.3 ng/dL measured 6 to 12 months after lobectomy and a Tg cutoff value of 11.0 ng/mL with normal TSH for the most recent measurement have been shown to be good predictors of the risk of recurrence (68).

### Follow-up after total thyroidectomy with or without radioiodine

The evaluation of the response to the initial treatment and the follow-up after TT are mainly based on Tg and TgAb levels. The values that define the response to treatment differ according to RAI treatment (Table 3).

The recurrence rates of patients with LRPTC classified as having an excellent response after the initial treatment is less than 2% (46). The follow-up of these patients can be limited to periodic (12 months) measurements of Tg and TgAb levels during levothyroxine therapy. The levothyroxine dose should be titrated to TSH levels in the normal or low range (0.5-2 IU/mL). After 5-10 years without evidence of disease, the risk of recurrence is so low that the need for routine assessments in specialized centers is debatable (69,70). Serum Tg measurements may be unable to identify small disease foci, particularly in the neck, which can be readily detected with neck US (71). However, the need for neck US monitoring is still a matter of debate since some studies have shown that the rate of false positive findings on neck US is high and may lead to inadequate treatment and/or follow-up (72,73). Furthermore, the effect of these early findings on patient outcomes has not been demonstrated, and the ATA guidelines recommend simple surveillance for suspicious lymph nodes with a < 10 mm (for lateral nodes) or < 8 mm (for central nodes) measurement in the short axis. We believe that in the absence of any other abnormalities in the first neck US, a subsequent US should not be scheduled before 3-5 years after the primary treatment, if ever (69,70).

An indeterminate response may be observed in 12%-23% of the patients with LRPTC, but 80%-90% of them will never experience disease recurrence (73,74). An analysis of stimulated Tg can be helpful in these cases. In patients with minimally increased stimulated Tg levels (< 2 ng/mL), Tg levels are likely to normalize spontaneously over time (73,74). Levels of Tg above 2 ng/mL are meaningful since the likelihood of persistent or recurrent disease increases with the increase in stimulated Tg levels. Up to 98% of the responses initially classified as indeterminate can be reclassified as excellent when stimulated Tg measurement is repeated 5 years after the initial treatment, compared with only 40% of the patients with an initial biochemical incomplete response (75). Also in these cases, the trend of Tg and TgAb values should be taken into consideration (44,75).

Only 10% of the patients with LRPTC treated with TT and RAI will show biochemical incomplete response (11). In this group of patients, serum Tg and TgAb levels should be monitored every 6-12 months, and neck US should also be performed yearly. Serial Tg measurements can be informative in these cases since Tg levels that are stable or decline over time indicate disease remission. Up to two thirds of these patients will later meet the criteria for an excellent response without any additional treatment (75-77). By contrast, serum Tg levels that rise over time are highly suspicious for persistent and/or recurrent disease (44,76) and should be explored with imaging studies.

In conclusion, the low mortality and recurrence/persistence rates of LRPTC allow for safe recommendations of different management approaches (Table 4). A multidisciplinary team can offer the best strategy considering the preferences of the patient, the experience of the medical team, and the characteristics associated with the patient’s quality of life.

**Table 4:** Follow up strategies based on response to initial therapys

Response to Therapy	Recommended follow-up
Excellent Response	Early decrease in the intensity and frequency of follow up and degree of TSH suppression Low and intermediate risk: Non stimulated Tg and TgAb every 12-24 months; Consider neck US in 12-24 months, if negative, no need for further imaging
Indeterminate Response	Decrease in the intensity and frequency of follow up and degree of TSH suppression Active surveillance: Serial imaging of non specific lesions; Non stimulated Tg and TgAb every 12-24 months
Biochemical Incomplete Response	Follow Tg curve with similar TSH levels. Stable or declining Tg levels = Active surveillance Non stimulated Tg and TgAb every 6-12 months, Stimulated Tg if clinically indicated; Neck US in 12 months. TSH suppression (mild) Increasing Tg or TgAb levels = Additional investigation Individualized approach with anatomic, functional and/or hybrid imaging. TSH suppression.
Structural Incomplete Response	Individualized approach: Consider actionable and non-actionable structural evidence of disease. TSH suppression
